# Hawk-Seq™ differentiates between various mutations in *Salmonella typhimurium* TA100 strain caused by exposure to Ames test-positive mutagens

**DOI:** 10.1093/mutage/geab006

**Published:** 2021-02-16

**Authors:** Yuki Otsubo, Shoji Matsumura, Naohiro Ikeda, Osamu Morita

**Affiliations:** 1 R&D Safety Science Research, Kao Corporation, 3-25-14 Tono-machi, Kawasaki-ku, Kawasaki City, Kanagawa 210–0821, Japan; 2 R&D Safety Science Research, Kao Corporation, 2606 Akabane, Ichikai-Machi, Haga-Gun, Tochigi 321–3497, Japan

## Abstract

A precise understanding of differences in genomic mutations according to the mutagenic mechanisms detected in mutagenicity data is required to evaluate the carcinogenicity of environmental mutagens. Recently, we developed a highly accurate genome sequencing method, ‘Hawk-Seq™’, that enables the detection of mutagen-induced genome-wide mutations. However, its applicability to detect various mutagens and identify differences in mutational profiles is not well understood. Thus, we evaluated DNA samples from *Salmonella typhimurium* TA100 exposed to 11 mutagens, including alkylating agents, aldehydes, an aromatic nitro compound, epoxides, aromatic amines and polycyclic aromatic hydrocarbons (PAHs). We extensively analysed mutagen-induced mutational profiles and studied their association with the mechanisms of mutagens. Hawk-Seq™ sensitively detected mutations induced by all 11 mutagens, including one that increased the number of revertants by approximately 2-fold in the Ames test. Although the sensitivity for less water-soluble mutagens was relatively low, we increased the sensitivity to obtain high-resolution spectra by modifying the exposure protocol. Moreover, two epoxides indicated similar 6- or 96-dimensional mutational patterns; likewise, three S_N_1-type alkylating agents indicated similar mutational patterns, suggesting that the mutational patterns are compound category specific. Meanwhile, an S_N_2 type alkylating agent exhibited unique mutational patterns compared to those of the S_N_1 type alkylating agents. Although the mutational patterns induced by aldehydes, the aromatic nitro compound, aromatic amines and PAHs did not differ substantially from each other, the maximum total base substitution frequencies (MTSFs) were similar among mutagens in the same structural groups. Furthermore, the MTSF was found to be associated with the carcinogenic potency of some direct-acting mutagens. These results indicate that our method can generate high-resolution mutational profiles to identify characteristic features of each mutagen. The detailed mutational data obtained by Hawk-Seq™ can provide useful information regarding mutagenic mechanisms and help identify its association with the carcinogenicity of mutagens without requiring carcinogenicity data.

## Introduction

Genomic mutations caused by environmental mutagens are associated with the aetiology of human cancers (1–3). These mutagens vary in their structure and mutagenic mechanism; each can cause a specific type of human cancer based on its structural and mutagenic characteristics. Importantly, the genomic mutations caused by different mutagens are distinct both in their pattern and quantity, which influence their carcinogenicity (4). Therefore, a better understanding of different mutational profiles along with mutagenic mechanisms would enable a more precise prediction of the carcinogenicity of environmental mutagens.

The mutagenicity of mutagens has been conventionally evaluated using mutagenicity assays, such as the Ames test (5). These assays can detect mutagen-induced mutations via surrogate marker genes with high sensitivity. Although these assays have improved our basic understanding of mutagenicity by measuring induced mutant frequencies via marker genes, they do not provide genome-wide mutation profiles, including the frequency or pattern of base substitutions (BSs). To better understand mutation profiles based on the mutagenic mechanism of mutagens, a method that provides mutation data with adequate resolution to identify characteristic features of each mutagen is required.

Recently, next-generation sequencing (NGS) technologies have enabled investigation of large-scale, genome-wide mutations (6,7). In cancer genomics, these technologies have helped characterise the landscape of genomic mutations in human cancer and improved the understanding of mutagenic traits in various cancer types (8–11). Although sequencing errors had hampered the detection of rare, mutagen-induced mutations, some studies achieved the detection of such mutations via the use of NGS (12–17). For example, Kucab *et al.* have reported a method to detect mutagen-induced mutations in isolated single cells (17). However, in this method, the results can be significantly influenced by the isolated clones studied. Meanwhile, sequencing methods using information from double-stranded DNA (dsDNA), able to detect mutations from numerous cells at once, have been shown to accurately detect these mutations (13,14). These NGS-based methods can thus differentiate between mutagen-induced genomic mutations with adequate resolution to identify characteristic features of mutagens.

We have previously reported a simple, highly accurate genome sequencing method named Hawk-Seq™ that used information from dsDNA (18). This method has the potential to improve the systemic understanding of mutation profiles for various mutagens by allowing high-throughput mutation analysis. The utility of this method was validated by acquiring data for five mutagens. However, to confirm whether it can expand our knowledge of mutagenicity, its use for detecting various types of mutagens should require detailed investigation. In addition, it has not been demonstrated whether Hawk-Seq™ can accurately identify genomic mutations to determine the characteristic features of mutagens based on their mutagenic mechanisms. Therefore, a detailed analysis of mutagen-induced mutations is required to validate the applicability and utility of Hawk-Seq™.

In the present study, we evaluated genomic DNA samples of *Salmonella typhimurium* TA100, which is widely used to detect base pair substitutions, exposed to various mutagens using Hawk-Seq™. We used several mutagens with different BS frequencies to determine the detection limit of Hawk-Seq™. In addition, a variety of structural groups were evaluated to study various mutation mechanisms. By scrutinising mutation data, such as mutation pattern, BS frequency and sequence context of mutations, we showed that Hawk-Seq™ can be used to study mutagenic mechanisms of mutagens as well as to determine its association with mutagenic carcinogenicity.

## Materials and methods

### Mutagens

1-methyl-3-nitro-1-nitrosoguanidine (MNNG; CASRN. 70-25-7), methyl methanesulfonate (MMS; CASRN. 66-27-3), 4-nitroquinoline N-oxide (4NQO; CASRN. 56-57-5), propylene oxide (PO; CASRN. 75-56-9), 2-acetamidofluorene (2AAF; CASRN. 53-96-3) and 7,12-dimethylbenz[a]anthracene (DMBA; CASRN. 57-97-6) were purchased from Tokyo Chemical Industry Co. Ltd. (Tokyo, Japan). Glyoxal (CASRN. 107-22-2) and glycidol (CASRN. 556-52-5) were purchased from FUJIFILM Wako Pure Chemical Corporation (Osaka, Japan). Formaldehyde (FA; CASRN. 50-00-0), 2-aminoanthracene (2AA; CASRN. 613-13-8) and 3-methylcholanthrene (3MC; CASRN. 56-49-5) were purchased from Sigma-Aldrich (St. Louis, MO, USA).

### Bacterial DNA sample preparation and Ames test

*S. typhimurium* Ames tester strain TA100 was obtained from the NITE Biological Resource Center (Tokyo, Japan). TA100 was pre-cultured for 4 h at 37°C in nutrient broth No. 2 (Oxoid, UK) containing 24 μg/ml ampicillin (Sigma-Aldrich, St. Louis, MO, USA). We measured the OD after bacterial pre-culture and confirmed that it was higher than 1.0. The obtained bacterial suspensions (OD > 1.0) were used to prepare genomic DNA samples for mutagen exposure, which was conducted in three independent tubes per dose according to the pre-incubation procedure of the standard Ames test with minor modifications of the DNA extraction protocol (5,15). Briefly, 100 µl of the above-described bacterial suspension was mixed with 500 µl of phosphate buffer, pH 7.4 (Nacalai Tesque, Kyoto, Japan) and 100 µl of dimethyl sulfoxide (DMSO) or test chemical solutions. For 2AA, 2AAF, DMBA and 3MC exposure, 500 µl of phenobarbital/5,6-benzoflavone-induced rat liver S9 mix (Kikkoman Biochemifa Company, Tokyo, Japan) was used instead of phosphate buffer. The resulting mixture was shaken at 100 rpm for 20 min at 37°C (pre-incubation step). Next, 50 µl of the mixture was inoculated into 2 ml nutrient broth containing ampicillin and agitated at 180 rpm for an additional 14 h at 37°C in the presence of mutagens to fix mutations. During the incubation of bacterial samples in the presence of mutagens that required metabolic activation, TA100 cells were inoculated in nutrient broth (NB) or broth containing 18.5% of the S9 mix (NB+S9); this concentration is identical to that in the soft agar used for the Ames test. After 14 h, genomic DNA was isolated using the DNeasy Blood and Tissue Kit (Qiagen, Valencia, CA, USA) according to the manufacturer’s instructions. Maximum doses for each mutagen were determined based on growth inhibition or solubility. For all mutagens, the Ames test was performed using a pre-incubation method with minimum glucose medium plates (Tesmedia^®^ AN; Oriental Yeast Co. Ltd., Tokyo, Japan). The plates were incubated at 37°C for 48 h, and the number of colonies was counted. For 3MC and DMBA, genomic DNA was extracted from plated cells after the Ames test as well as from suspension culture samples.

### Library preparation and sequencing

TA100 genomic DNA samples were sheared to fragments with a peak size of 350 bp using a sonicator (Covaris, Woburn, MA, USA). The obtained DNA fragments were used for library construction using the TruSeq nano DNA library preparation kit (TruSeq; Illumina, San Diego, CA, USA), with a slight modification for Hawk-Seq™. Briefly, DNA fragments were subjected to end repair, 3′ dA-tailing and ligation to TruSeq-indexed adaptors according to the manufacturer’s instructions. Thereafter, the DNA concentration of each sample was measured using Agilent 4200 TapeStation (Agilent Technologies, Santa Clara, CA, USA). Ligated products were diluted with suspension buffer, and 78 amol of the ligated products were PCR amplified (18). The amplified PCR products were sequenced with 2 × 100 bp or 2 × 150 bp to yield ca. 50 M read pairs using HiSeq2500 or HiSeqX (Illumina, San Diego, CA, USA).

### Data processing for Hawk-Seq^TM^

Adaptor sequences and low-quality bases were removed from the generated read pairs using Cutadapt-1.16 (19). The resulting paired-end reads were mapped to reference genome sequences to prepare a SAM file using Bowtie2-2.3.4.1 (20). *S. typhimurium* LT-2 genome (GCA000006945.2) was used as the reference genome sequence. SAM processing was performed using SAMtools-1.7 (21). The double-strand DNA consensus sequence (dsDCS) was prepared according to the Hawk-Seq™ method (18). Briefly, read pairs that shared the same genomic location were grouped into the same position groups and divided into two subgroups according to their orientation. The same position groups that included read pairs in both read directions were used to generate dsDCS read pairs. These dsDCS read pairs were mapped again to the reference genome sequence using Bowtie2. The resulting SAM files were processed using SAMtools, and mutations were detected.

### Mutation detection and statistical analyses

To calculate the BS frequency, the number of BSs for each type was enumerated. The BS frequencies for each mutation type per 10^6^ G:C or A:T base pairs were calculated by dividing the mutation count by the total read base count mapped to the G:C or A:T base pairs. Statistical analyses were performed based on the frequency of each mutation type per 10^6^ bp using Dunnett’s multiple comparison test or Student’s *t*-test. Known variant positions of TA100 were removed from the analysis (22). During the analysis of genomic samples extracted from cells in minimum glucose agar, the positions with three or more identical mutations were not included because these mutations were considered to originate from an identical mutation shared by cells of the same colony. To determine the 96-dimensional mutation patterns (trinucleotide mutational signatures) that include BS frequencies by neighbouring bases, the bases flanking the 5′ and 3′ of each mutated residue were analysed. Moreover, the BS frequencies were calculated within the context of each trinucleotide. The similarities between them and COSMIC single BS (SBS) signatures were evaluated based on cosine similarity (CS) (23). Principal component analysis (PCA) was performed using the data of 6-dimensional mutational spectra (G:C > T:A, G:C > C:G, G:C > A:T, A:T > T:A, A:T > C:G and A:T > G:C) (24). Individual features in the six types of mutation spectra were standardised. PC scores and the proportion of variances of each PC were subsequently calculated.

### Calculation of total BS frequency and analysis of relationship with carcinogenic potency

To determine the relationship between chemical structures and mutational features obtained by Hawk-Seq™, the total BS frequency was calculated by dividing the number of all BSs by the total read base count mapped to G:C and A:T base pairs. Furthermore, the association of BS frequency with carcinogenic potency was determined using TD50 values (mutagen concentration at which tumour growth is observed in 50% of tested animals) in the Carcinogenicity Potency Database (CPDB) (25). Individual chemicals in CPDB were assigned multiple TD50 values (mg/kg/day) based on the results of multiple carcinogenicity tests. We then analysed the correlation between the maximum value of total BS frequency and the lowest available potency value, indicating the most sensitive TD50 (26).

## Results

### Selection of mutagens

To understand the applicability and utility of Hawk-Seq™, we selected 11 mutagens (MNNG, MMS, Glyoxal, FA, 4NQO, Glycidol, PO, 2AA, 2AAF, 3MC and DMBA). These mutagens have diverse chemical structures and consist of alkylating agents, aldehydes, an aromatic nitro compound, epoxides, aromatic amines and polycyclic aromatic hydrocarbons (PAHs) (27,28). The results of the Ames test using TA100 showed that the maximum number of His^+^ revertants generated by these mutagens is variable (Table 1; Supplementary Figure S1). Therefore, we used these mutagens to study the applicability and utility of Hawk-Seq™ by elucidating differences in the mutagenic characteristics based on their mechanism.

**Table 1.** Results of the Ames test

Structural group	Chemical	Dose(µg/plate)	Revertants/plate (*n* = 3)		*n*-fold increase	CV
			Mean	SD		
Alkylating agents	MNNG	0	122.0	13.9	–	–
		1	154.3	3.1	1.27	0.020
		5	2115.0	358.6	17.3	0.170
	MMS	0	116.0	25.2	–	–
		30	222.7	28.9	1.92	0.130
		1000	1956.3	116.2	16.9	0.059
Aldehydes	Glyoxal	0	116.0	25.2	–	–
		30	472.3	45.5	4.07	0.096
		60	900.0	73.0	7.76	0.081
	FA	0	133.7	9.6	–	–
		10	205.0	17.6	1.53	0.086
		20	223.3	7.4	1.67	0.033
Aromatic nitro compounds	4NQO	0	133.7	9.6	–	–
		0.1	851.7	41.8	6.37	0.049
		0.5	2181.7	263.6	16.3	0.121
Epoxides	Glycidol	0	134.3	9.5	–	–
		1000	2252.3	220.7	16.8	0.098
		10 000	6492.7	936.9	48.3	0.144
	PO	0	91.0	11.0	–	–
		5000	1363.0	92.8	15.0	0.068
		10 000	1655.3	20.6	18.2	0.012
Aromatic amines	2-AA	0	112.3	7.5	–	–
		5	1265.0	154.3	11.3	0.122
		7	3131.3	141.9	27.9	0.045
	2-AAF	0	112.3	7.5	–	–
		40	1464.3	761.3	13.0	0.520
		80	3903.7	108.6	34.8	0.028
		120	4276.3	710.0	38.1	0.166
PAHs	3MC	0	100.7	10.0	–	–
		20	1510.7	27.5	15.0	0.018
		100	1618.3	155.1	16.1	0.096
	DMBA	0	100.7	10.0	–	–
		10	707.3	23.2	7.03	0.033
		20	1499.7	184.1	14.9	0.123
		100	853.0	64.6	8.47	0.076

### Detection of mutagenicity induced by direct-acting mutagens

**Figure 1.**
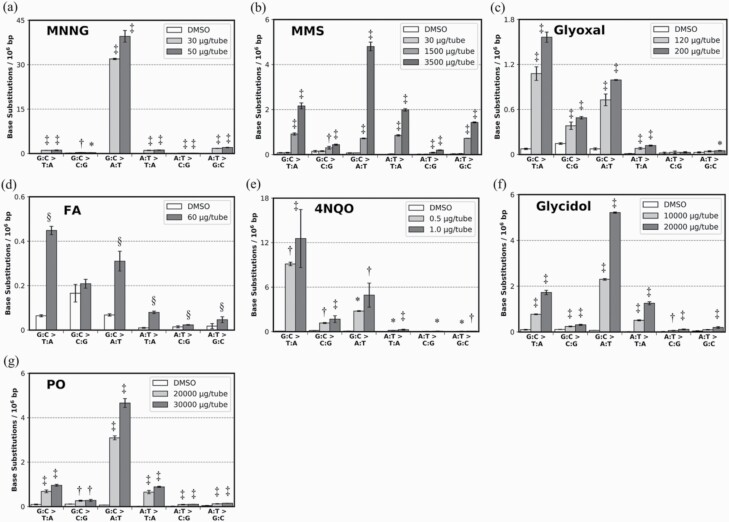
Mutational spectra induced by (**a**) MNNG (30, 50 µg/tube), (**b**) MMS (30, 1500, 3500 µg/tube), (**c**) glyoxal (120, 200 µg/tube), (**d)** FA (60 µg/tube), (**e**) 4NQO (0.5, 1 µg/tube), (**f**) glycidol (10 000, 20 000 µg/tube) and (**g**) PO (20 000, 30 000 µg/tube) in TA100 cells. The BS frequencies per 10^6^ G:C or A:T base pairs are displayed (*n* = 3). Error bars represent standard deviation. Asterisks and daggers indicate *P* values (Dunnett’s multiple comparison test: *P < 0.05, ^†^*P* < 0.01 and ^‡^*P* < 0.001; Student’s *t* test: ^§^*P* < 0.05).

First, we evaluated mutagens that do not require metabolic activation: MNNG, MMS, glyoxal, FA, 4NQO, glycidol and PO. After mutagen exposure, the OD660 values of bacterial samples were substantially decreased at the maximum dose (Supplementary Table SIa and b), indicating adequate exposure of the bacterial cells to mutagens. During the analysis of samples treated with these mutagens (*n* = 54), 58 ± 5.9 M read pairs per sample were subjected to Hawk-Seq™, and 4.8 ± 0.5 dsDCS read pairs per sample were obtained. The mean frequency of each BS in the DMSO-treated samples (*n* = 12) were 0.077 × 10^–6^ bp on G:C > T:A mutation, 0.14 × 10^–6^ bp on G:C > C:G mutation, 0.069 × 10^–6^ bp on G:C > A:T mutation, 0.0095 × 10^–6^ bp on A:T > T:A mutation, 0.015 × 10^–6^ bp on A:T > C:G mutation and 0.028 × 10^–6^ bp on A:T > G:C mutation. Mutations induced by these mutagens were detected by Hawk-Seq™ (Figure 1a–g). The mutational pattern induced by alkylating agents, such as MNNG, was predominantly G:C > A:T; these mutations were observed at 39.6 × 10^–6^ base pairs (bp) in samples treated with 50 µg/tube MNNG (Figure 1a). In MMS-treated samples, the most frequent G:C > A:T mutational pattern were observed at 4.80 × 10^–6^ bp. Moreover, G:C > T:A and A:T > G:C mutations were observed as minor patterns in these samples (Figure 1b). These results are consistent with those reported previously (29,30). Furthermore, a previously unreported A:T > T:A mutation was detected as a minor pattern in MMS-treated samples. Glyoxal, FA and 4NQO showed similar mutational spectra and induced G:C > T:A mutation at 1.56 × 10^–6^ bp, 0.45 × 10^–6^ bp and 12.5 × 10^–6^ bp, respectively, in the highest dose group (Figure 1c–e). These results were also consistent with those in previous studies (17,30–32). Other minor mutations, such as G:C > C:G in 4NQO-treated samples and A:T > T:A in FA-treated samples, were also detected; these mutations have not been reported previously. The two epoxides—glycidol and PO—induced G:C > T:A, G:C > A:T and A:T > T:A mutations, and their mutation spectra were highly similar (Figure 1f and g). The most frequent G:C > A:T mutation pattern was observed at 5.21 × 10^–6^ and 4.66 × 10^–6^ bp in glycidol- and PO-treated samples, respectively. This represents the first characterisation of the mutational pattern induced by glycidol, which has not been previously reported. Meanwhile, regarding the mutational patterns induced by PO, the G:C > A:T and A:T > T:A mutations were reported previously (17), whereas the G:C > T:A mutation was not. Among all 11 mutagens, FA-induced samples had the least number of revertants in the Ames test, which indicated that the number of revertants were approximately 2-fold higher than that in control samples. However, G:C > T:A frequency in FA-treated samples was 6.97-fold higher than that in the controls in Hawk-Seq™ analysis (Supplementary Table SII). These results suggest that Hawk-Seq™ can detect mutagen-induced mutations with high sensitivity.

### Detection of mutations induced by mutagens that require metabolic activation

**Figure 2.**
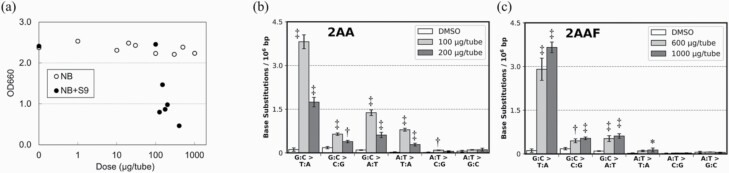
Analyses of aromatic amine mutagens. (**a**) OD660 values for TA100 cell suspensions cultured for 14 h in NB (white circles) or NB+S9 (black circles) after 2AA exposure are shown. The growth of TA100 cells cultured in NB+S9 was significantly inhibited. BS frequencies in TA100 cells cultured in NB+S9 after (**b**) 2AA (100, 200 µg/tube) or (**c**) 2AAF (600, 1000 µg/tube) exposure. The BS frequencies per 10^6^ G:C or A:T base pairs are displayed (*n* = 3). Error bars represent standard deviation. Asterisks and daggers indicate *P* values (Dunnett’s multiple comparison test; *P < 0.05, ^†^*P* < 0.01 and ^‡^*P* < 0.001).

For the analysis of mutagens requiring metabolic activation (2AA, 2AAF, 3MC and DMBA), TA100 was incubated in NB+S9 following pre-incubation with the test samples since no clear growth inhibition was observed after test samples were treated with 2AA in NB (Figure 2a, white circles). Meanwhile, substantial cytotoxicity was observed when the test samples were treated with 2AA in NB+S9 (Figure 2a, black circles; Supplementary Table SIc). The BS frequency in 2AA-treated samples was also increased in NB+S9 compared to that in NB (Supplementary Figure S2). In the NB+S9 condition, the G:C > T:A mutation was observed at 3.82 × 10^–6^ and 3.66 × 10^–6^ bp in 2AA- and 2AAF-treated samples, respectively. The G:C > A:T mutations were induced in 2AA- and 2AAF-treated samples at frequencies of 0.70 × 10^–6^ and 0.67 × 10^–6^ bp, respectively. Meanwhile, the A:T > T:A mutation was induced at 0.33 × 10^–6^ bp in 2AA-treated samples. 2AA and 2AAF are known to induce these characteristic mutations (33,34) (Figure 2b and c). A previously unreported G:C > C:G mutation was also detected in 2AA- and 2AAF-treated samples at frequencies of 0.40 × 10^–6^ and 0.58 × 10^–6^ bp, respectively.

**Figure 3.**
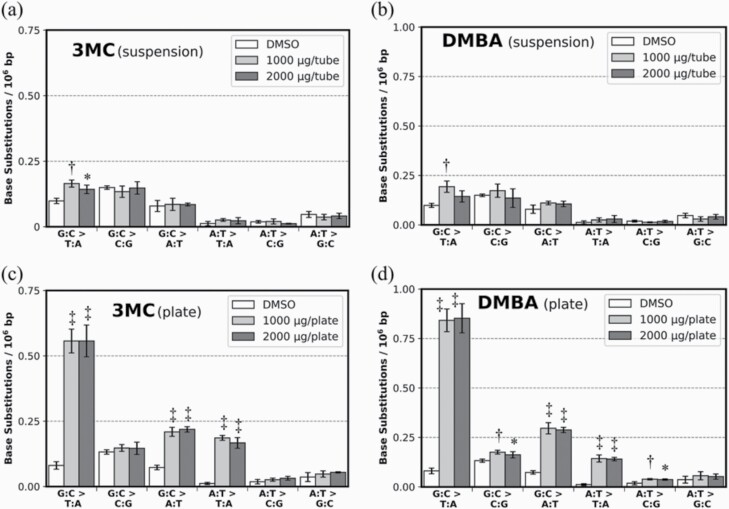
Enhancement of PAH-induced mutation sensitivity by modifying exposure to mutagens. BS frequencies in TA100 cells cultured in NB+S9 after (**a**) 3MC (1000, 2000 µg/tube) and (**b**) DMBA (1000, 2000 µg/tube) exposure. BS frequencies in TA100 cells cultured for 48 h on minimum glucose medium plate after (**c**) 3MC (1000, 2000 µg/plate) and (**d**) DMBA (1000, 2000 µg/plate) exposure. The BS frequencies per 10^6^ G:C or A:T base pairs are displayed (*n* = 3). Error bars represent standard deviation. Asterisks and daggers indicate *P* values (Dunnett’s multiple comparison test; *P < 0.05, ^†^*P* < 0.01 and ^‡^*P* < 0.001).

Meanwhile, no cytotoxicity was observed in 3MC- and DMBA-treated TA100 samples, which were prepared using the same protocol as for 2AA- and 2AAF-treated TA100 samples (Supplementary Table SId). No cytotoxicity was observed even at the higher concentrations of S9 mix (30% and 50% in NB; data not shown). A slight increase in G:C > T:A frequencies was observed in these samples (Figure 3a and b). The high LogKow value of PAHs (Supplementary Table SIII) indicates that they are highly water insoluble (35); therefore, these mutagens might not be able to induce mutations in TA100 under suspension culture conditions. We identified genomic DNA mutations in cells plated on agar after the Ames test because mutagen exposure substantially increased the number of revertants in the Ames test. Consequently, we primarily detected an increase in G:C > T:A mutation as in the suspension culture samples. However, in these samples, the induction of G:C > T:A mutation, compared to controls, was increased by 4.1- and 5.4-fold compared to those in suspension culture. The G:C > T:A mutation frequencies in 3MC- and DMBA-treated samples on agar plates were 0.62 × 10^–6^ bp and 0.88 × 10^–6^ bp, respectively (Figure 3c and d). 3MC and DMBA also induced previously reported minor mutation, such as G:C > A:T and A:T > T:A (36,37). This procedure requires a few more processing steps than the procedure in which mutagens are exposed to cells in suspension cultures. However, this method can enable the analysis of multiple cells plated on agar.

### Trinucleotide mutational signatures

**Figure 4.**
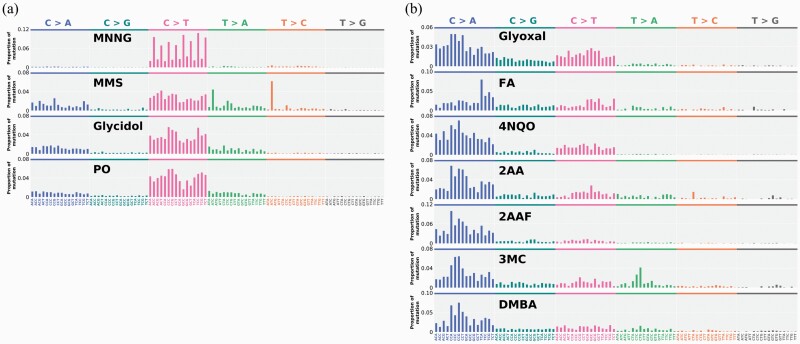
The 96-dimensional mutation pattern in TA100 cells after mutagen exposure. The mean proportion of BS frequencies for each trinucleotide in cells exposed to MNNG (50 µg/tube), MMS (3500 µg/tube), glycidol (20 000 µg/tube), PO (30 000 µg/tube), glyoxal (200 µg/tube), FA (60 µg/tube), 4NQO (1.0 µg/tube), 2AA (100 µg/tube), 2AAF (1000 µg/tube), 3MC (1000 µg/plate) and DMBA (1000 µg/plate) are shown.

The 96-dimensional trinucleotide mutational pattern has been proven to be effective for the identification of characteristic mutation patterns, representing mutagenic mechanisms of each mutagen, in human cancer genomics (23,38), and mutagen-induced mutation analysis (12,17,18). We analysed the sequence context of mutations in samples treated with 11 mutagens and generated 96-dimensional mutational patterns. MNNG-treated samples showed G:C > A:T mutations at ‘NpCpY’ (where Y indicates a pyrimidine base; Figure 4a), analogous to typical alkylating agents, such as ethylnitrosourea (ENU) and methylnitrosourea (MNU) treated TA100 samples (18). The G:C > A:T mutational pattern in MMS-treated samples was different from that in MNNG-treated samples. The mutational patterns induced by glycidol and PO exhibited a high CS value: 0.98. In the 6-dimensional mutational pattern analysis, the patterns induced by glycidol and PO were similar to those induced by MMS, except that the A:T > G:C mutation was induced only in MMS-treated samples. However, the 96-dimensional mutation patterns of MMS-treated samples showed distinct peaks, representing the A:T > T:A and A:T > G:C mutations at ‘ApTpC’, while those of epoxides-treated samples did not. These results indicated that the mutagenesis sequence context dependency should also be considered for each mutagen. In contrast, the 96-dimensional mutational patterns of glyoxal-, 4NQO-, 2AA-, 2AAF-, 3MC- and DMBA-treated samples also showed similar broad spectra for G:C > T:A (Figure 4b). To specifically compare their context dependencies on the G:C > T:A mutation, we calculated the CS limited to 16 dimensions on G:C > T:A mutations among these mutagens. The CS values for the 16-dimensional G:C > T:A mutational pattern induced by these mutagens were greater than 0.90. However, the CS values for the 16-dimensional G:C > T:A mutational pattern induced by FA and other mutagens were relatively low (each value was less than 0.80).

We then compared the 96-dimensional mutation patterns induced by 11 mutagens with 49 SBS signatures in COSMIC (23) (Supplementary Figure S3). The pattern induced by MNNG showed a considerably high CS (0.96) with SBS11 (alkylating agent). Meanwhile, the pattern induced by MMS did not show high similarity with any signatures in COSMIC (a CS value of 0.65 with SBS32 was the highest). The highest CS values for the mutational patterns of glycidol- and PO-treated samples with SBS30 were 0.77 and 0.79, respectively. The mutational patterns of 4NQO-, glyoxal-, 2AA-, 2AAF-, DMBA- and 3MC-treated samples showed CS values greater than 0.80 compared to that of SBS4 (smoking tobacco).

### PCA based on mutational spectra

**Figure 5.**
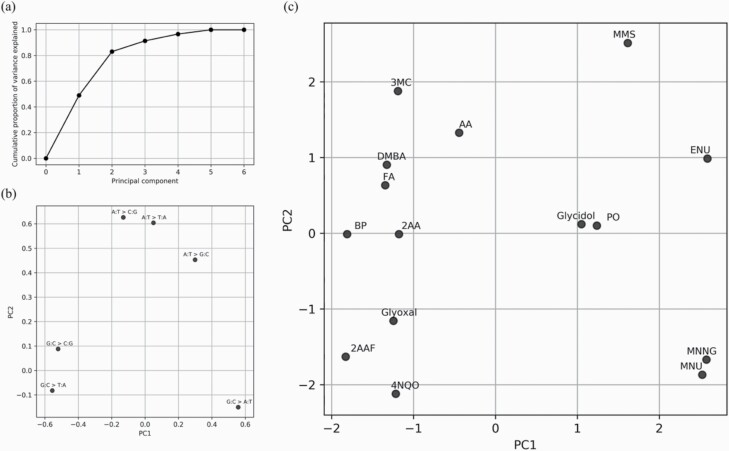
PCA results using the 6-dimensional mutation spectra for each mutagen. (**a)** Cumulative proportion of the variance of each principal component (PC1–PC6). These values are added up to 1. (**b**) PCA loading of each base substitution type on PC1 and PC2. The PC1 score correlates positively with G:C > A:T and A:T > G:C and negatively with G:C > T:A and G:C > C:G. The PC2 score correlates positively with mutations on the A:T base pair. (**c**) The PCA score plot for each mutagen is based on their PC1 and PC2 values.

We have previously obtained mutational profiles of TA100 cells that were exposed to ENU, MNU, aristolochic acid I (AA) and benzo[a]pyrene (BP) (18). Here, we performed PCA using the 6-dimensional mutational spectra of 15 mutagens, including the abovementioned four mutagens (Figure 5a–c). The proportions of variance for PC1 and PC2 were 0.50 and 0.36, respectively (Figure 5a). The PC1 values correlated positively with the ratio of G:C > A:T and negatively with that of G:C > T:A, whereas the PC2 values correlated positively with the ratios of A:T base pair mutations (Figure 5b). We visualised the relationships of the 15 mutagens by plotting them based on their PC1 and PC2 values and obtained an outcome reflecting their mutagenic mechanisms (Figure 5c). For example, among the four alkylating agents, the two *N*-methyl-*N*-nitroso compounds—MNNG and MNU—plotted closely with each other but were distant from ENU and MMS. Although two epoxides (glycidol and PO) showed similar mutation patterns to MMS, they were plotted closely with each other but distantly from MMS. Moreover, other mutagens that form bulky DNA adducts were distantly located from alkylating agents and epoxides. These results indicate that the 6-dimensional mutation spectra can be used to differentiate between mutagens based on their mechanisms and provide insights into their classification. A similar plot based on PCA was obtained using data generated from the 96-dimensional mutation pattern of 15 mutagens (Supplementary Figure S4).

### Total BS frequency and its association with carcinogenic potency

**Figure 6.**
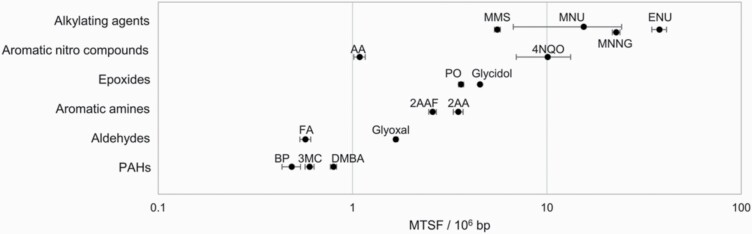
The maximum total BS frequency induced by each mutagen is shown. The total BS frequencies per 10^6^ base pairs are displayed in log-scale (*n* = 3). Error bars represent standard deviation.

Using Hawk-Seq™ analysis data, we found that the maximum total BS frequencies (MTSFs) of chemicals with similar structural groups were comparable (Figure 6). MTSF is the maximum value of the total BS frequency observed in samples exposed to several doses of individual mutagens. For example, alkylating agents have relatively high MTSFs, whereas aldehydes and PAHs have low MTSFs. Importantly, this parameter provides information about the mutagenic mechanism of mutagens. To investigate the relationship between this parameter and the carcinogenic potency, we evaluated the correlation between MTSF and TD50 values of seven direct-acting mutagens present in CPDB. We used this approach because the S9 mix might not accurately mimic *in vivo* metabolism. MTSF showed a considerable negative correlation with TD50 values for all mutagens, except FA (Supplementary Figure S5a and b). Mutagens with high carcinogenic potency (ENU, MNU and MNNG), which are considered cohort of concern in the threshold of toxicological concern concept (39), showed high MTSF values. Less potent carcinogens, such as MMS, glycidol and PO, showed relatively lower MTSF than ENU, MNU and MNNG. We also determined the BS frequency per dose and analysed the correlation between them and TD50 values (Supplementary Figure S6a and b). However, less association was observed with structural category and TD50 values compared to MTSF.

## Discussion

Herein, we demonstrated that Hawk-Seq™ can sensitively detect mutations induced by all evaluated mutagens. The increase in the Hawk-Seq™ BS frequency induced by most of the 11 mutagens was equivalent to, or greater than, that in the number of His^+^ revertants in the Ames test, suggesting that Hawk-Seq™ generally provides a more sensitive and stable evaluation of mutagenicity than the Ames test. In particular, the maximum *n*-fold increase in the number of revertants in FA-treated samples was 1.67, and the coefficient of variation (CV) for this group was 0.033. Using Hawk-Seq™, the frequency of G:C > T:A increased by 6.97-fold in FA-treated samples, and the CV for this mutation pattern was 0.042. The Ames assay can only detect viable, phenotypically expressed mutants capable of sustained growth, while Hawk-Seq™ detects variants without the need for sustained growth. The extensive DNA-protein cross-linking effects of chemicals, such as FA, may explain the differential sensitivity of the two methods. For PAH-treated samples, the *n*-fold increase in the BS frequency was relatively smaller than that in the number of revertants. This was partly because the error rate for the G:C > T:A mutation—the predominant mutation type induced by PAHs—in the control was relatively higher than that for other mutation types (40,41). Therefore, mutations induced by these mutagens would be detected more sensitively by decreasing the number of errors. In addition, the exposure conditions, including the duration of mutagen exposure or the concentration of S9 mix, have not been optimised. Therefore, Hawk-Seq™ might be able to detect BSs more sensitively by optimising experimental conditions.

Regarding mutagens requiring metabolic activation, the cytotoxicity was not promoted by extending the length of pre-incubation (1, 2, 3, and 4 h; data not shown). Thus, as indicated in our results, metabolic activation after the pre-incubation step is required to sensitively detect mutations. Meanwhile, the sensitivity for very low water-soluble mutagens, such as PAHs, was increased by analysing cells, including His^+^ revertants on agar plates. Because genomic DNA was extracted from cells with revertants, we were concerned that the results included bias for the detected mutations. However, the 96-dimensional mutation patterns of cells grown on agar plates and treated with 3MC and DMBA showed high similarity with that of TA100 cells grown in suspension and treated with BP (18). These results suggest that the selection of cells on agar plates does not clearly cause bias in specific context.

The high-resolution data, including minor mutational patterns, can be used to understand the similarities or differences between the mutagenic mechanisms of mutagens. For example, 6- and 96-dimensional mutational patterns for two epoxides were highly similar. In addition, the mutational patterns of MNNG-, MNU- and ENU-treated samples (all S_N_1-type alkylating agents) were similar (42). Although S_N_2-reactive MMS induced G:C > A:T mutation, similar to the S_N_1-type alkylating agents, it showed different mutational patterns for other mutations. PCA using the mutational spectra data clearly distinguished these mutagens based on their mechanisms. Thus, the mutagenic mechanism of each mutagen based on its reactive group, such as alkylating agent or epoxide, was detected in the mutational patterns. Additionally, the MTSF values of mutagens with identical structural groups were comparable; therefore, these values can help to better understand the mutagenic mechanisms of mutagens. Meanwhile, there were no clear differences in the mutational patterns between glyoxal-, FA-, 4NQO-, 2AA-, 2AAF-, 3MC- and DMBA-treated samples. Because the bacterial genome is simpler in epigenetic regulation than the mammalian genome, the mutation pattern did not identify differences in the mechanisms of these mutagens. For example, BP induced more G:C mutations in the CpG context in mice (18); however, this tendency was not observed in TA100 cells. The occurrence of CpG methylation is thought to be responsible for this disparity (43). Therefore, it might be useful to evaluate various mutagens in mammalian cell systems. Another possible cause of this difference is the unique drug-metabolism systems within rodents and S9 used in the Ames test. However, the mutational data of various mutagens on various biological models would be required.

Previously, efforts to find a correlation between the results of Ames test and carcinogenic potency have not provided desirable outcomes (44). This is partly due to the limitation of indirect evaluation via surrogate markers; the reversion mutations reflect only a small number of mutations in large genomes. Meanwhile, Hawk-Seq™ provides a mutational landscape across the whole genome. Although the number of mutagens is limited, we found that the MTSF across the whole genome negatively correlated with the most sensitive TD50 values in CPDB. Because FA can induce DNA-protein crosslinks (45) and double-strand breaks under toxic conditions (46), the data regarding only BS mutations did not sufficiently reflect the carcinogenic potency of FA. These results suggest that the carcinogenic potency of mutagens, which induce BS as a major mutagenic mechanism, could be estimated from MTSF values. Furthermore, only six mutagens were evaluated in this correlation analysis; therefore, further investigations need to be conducted using more mutagens.

In conclusion, this study demonstrated that Hawk-Seq™ can sensitively detect a wide range of mutagens and can provide high-resolution mutation data to identify the characteristic features of each category of mutagens. A thorough analysis of the mutational data can provide useful information regarding mutagenic mechanisms and their association with carcinogenicity.

## Supplementary Material

geab006_suppl_Supplementary_Material

## Data Availability

*S. typhimurium* strain sequence data used in this study are available at the DNA Data Bank of Japan Sequence Read Archive under accession number DRA010740.
