# Supplementary material for: Hawk-Seq™ differentiates between various mutations in Salmonella typhimurium TA100 strain caused by exposure to Ames test-positive mutagens
Source: Mutagenesis. 2021 Feb 16;36(3):245–54. doi: 10.1093/mutage/geab006 (PMC8262380; doi:10.1093/mutage/geab006)
Supplement: geab006_suppl_Supplementary_Material [file geab006_suppl_supplementary_material.pdf]

SUPPLEMENTARY DATA

Hawk-Seq™ differentiates between various mutations in *Salmonella typhimurium* TA100 strain caused by exposure to Ames test-positive mutagens

Yuki Otsubo, Shoji Matsumura, Naohiro Ikeda, and Osamu Morita

Table SI. OD660 values of suspension cultures measured after 14-h mutagen exposure. The samples were treated with **a, b** direct-acting mutagens without metabolic activation, or **c, d** mutagens in NB and in NB+S9, following pre-incubation.

(a)

Chemical	Dose (μg/tube)	Sample No.	OD660
MNNG	0	1	2.393
		2	2.405
		3	2.461
	30	1	2.09
		2	2.055
		3	2.032
	50	1	0.415
		2	0.41
		3	0.549
MMS	0	1	2.483
		2	2.364
		3	2.398
	30	1	2.331
		2	2.41
		3	2.452
	1500	1	2.195
		2	2.107
		3	2.224
	3500	1	0.765
		2	0.629
		3	0.622
Glyoxal	0	1	2.441
		2	2.437
		3	2.429
	120	1	2.384
		2	2.276
		3	2.179
	200	1	0.521
		2	0.71
		3	0.672

(b)

Chemical	Dose (μg/tube)	Sample No.	OD660
FA	0	1	2.505
		2	2.447
		3	2.367
	60	1	0.45
		2	0.372
		3	0.403
4NQO	0	1	2.505
		2	2.447
		3	2.367
	0.5	1	2.287
		2	2.33
		3	2.273
	1	1	0.444
		2	0.434
		3	0.579
Glycidol	0	1	2.474
		2	2.452
		3	2.517
	10000	1	2.047
		2	2.047
		3	2.029
	20000	1	0.821
		2	0.594
		3	0.834
PO	0	1	2.477
		2	2.425
		3	2.432
	20000	1	2.054
		2	2.13
		3	2.095
	30000	1	0.395
		2	0.169
		3	0.413

(c)

Chemical	Dose (µg/tube)	Sample No.	OD660
2AA (NB+S9)	0	1	2.599
		2	2.616
		3	2.535
	100	1	2.56
		2	2.315
		3	2.538
	200	1	1.021
		2	0.717
		3	1.206
2AA (NB)	0	1	2.384
		2	2.351
	200	1	2.215
		2	2.176
2AAF (NB+S9)	0	1	2.599
		2	2.616
		3	2.535
	600	1	1.761
		2	1.611
		3	1.395
	1000	1	0.993
		2	0.938
		3	1.143

(d)

Chemical	Dose (µg/tube)	Sample No.	OD660
3MC (NB+S9)	0	1	2.637
		2	2.569
		3	2.618
	1000	1	2.605
		2	2.589
		3	2.689
	2000	1	2.734
		2	2.648
		3	2.696
DMBA (NB+S9)	0	1	2.637
		2	2.569
		3	2.618
	1000	1	2.553
		2	2.631
		3	2.618
	2000	1	2.565
		2	2.571
		3	2.619

In the dose column, 0 mg/mL represents the vehicle control (i.e. DMSO).

Table SII. Comparison of sensitivity and robustness between Ames test and Hawk-Seq™. The n-fold increase and CV for the number of His^+ revertants or mutation frequencies are presented.

Mutagen	Ames test		Hawk-Seq™		
	n-fold increase	CV	n-fold increase	CV	Major spectrum
MNNG	17.3	0.170	582.2	0.050	G:C > A:T
MMS	16.9	0.059	71.8	0.040	G:C > A:T
Glyoxal	7.76	0.081	21.1	0.044	G:C > T:A
FA	1.67	0.033	6.97	0.042	G:C > T:A
4NQO	16.3	0.121	169.3	0.312	G:C > T:A
Glycidol	48.3	0.144	78.5	0.005	G:C > A:T
PO	18.2	0.012	70.2	0.044	G:C > A:T
2-AA	27.9	0.045	34.3	0.063	G:C > T:A
2-AAF	38.1	0.166	32.9	0.050	G:C > T:A
3MC	16.1	0.096	6.91	0.081	G:C > T:A
DMBA	14.9	0.123	10.4	0.068	G:C > T:A

Table SIII. LogKow value for mutagens with low water solubility.

Chemical	LogKow*
2AA	3.12
2AAF	3.43
3MC	6.13
DMBA	6.42

* Values were calculated using KOWWIN ver. 1.68.

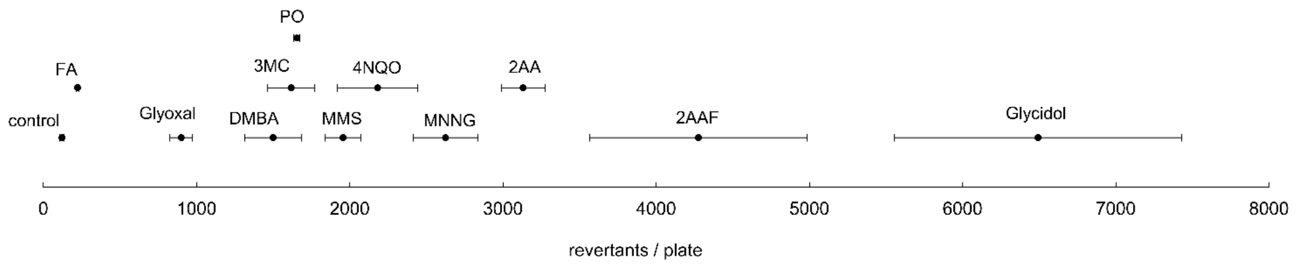

Fig. S1. Maximum number of mutagen-induced His^+ revertants (n = 3). Error bars represent standard deviation.

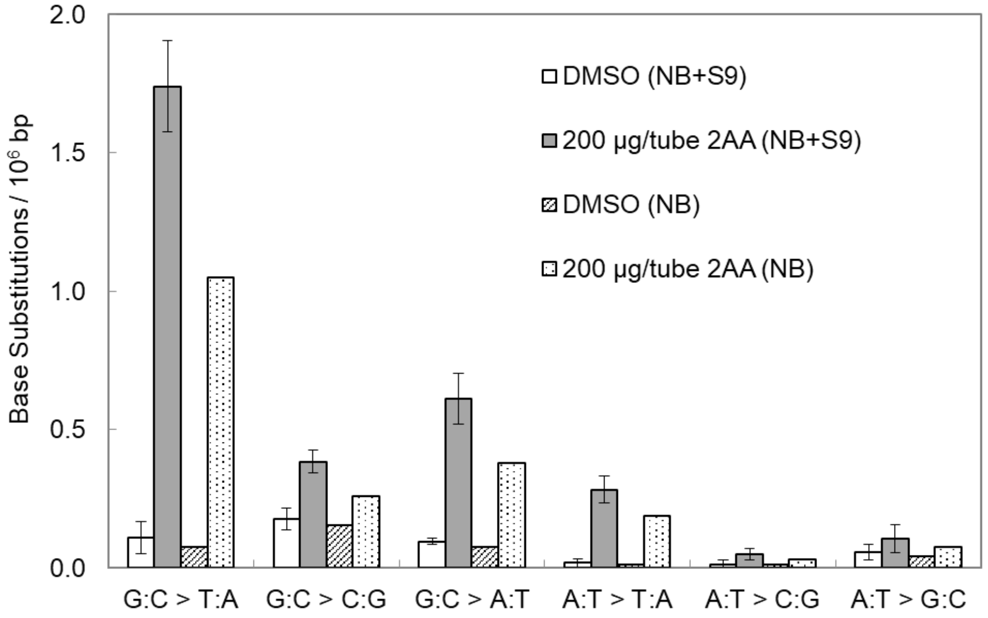

Fig. S2. BS frequencies induced by 2AA (200 µg/tube) in TA100 in NB (n = 2) and NB+S9 (n = 3) during the after pre-incubation. The BS frequencies per 10^6 G:C or A:T base pairs are shown. Error bars represent standard deviation.

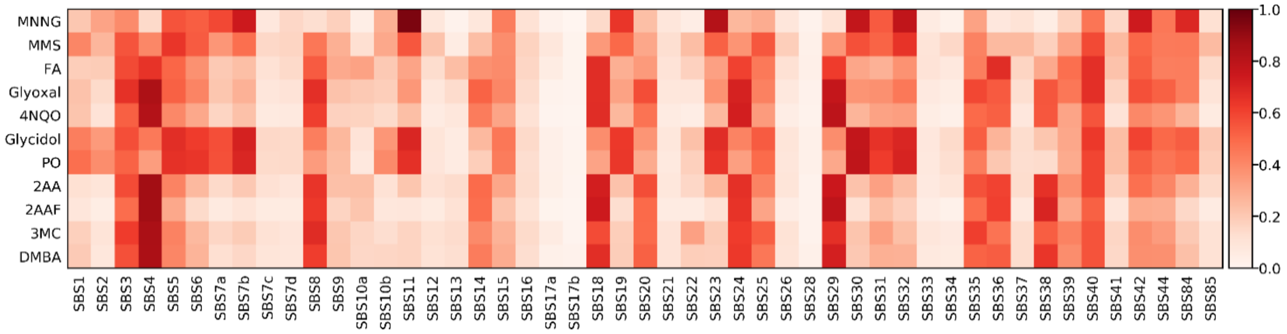

Fig. S3. Relationship between human cancer and mutagens. Recent somatic mutations from tens of thousands of human cancer samples were accumulated; refined analysis identified 49 signatures formatted by 96 types of trinucleotides from the HUGE database [Alexandrov, 2020]. To evaluate the similarity between each 96-trinucleotide mutation pattern, the cosine similarities (CS) between signatures of mutagens or signatures listed in COSMIC were calculated.

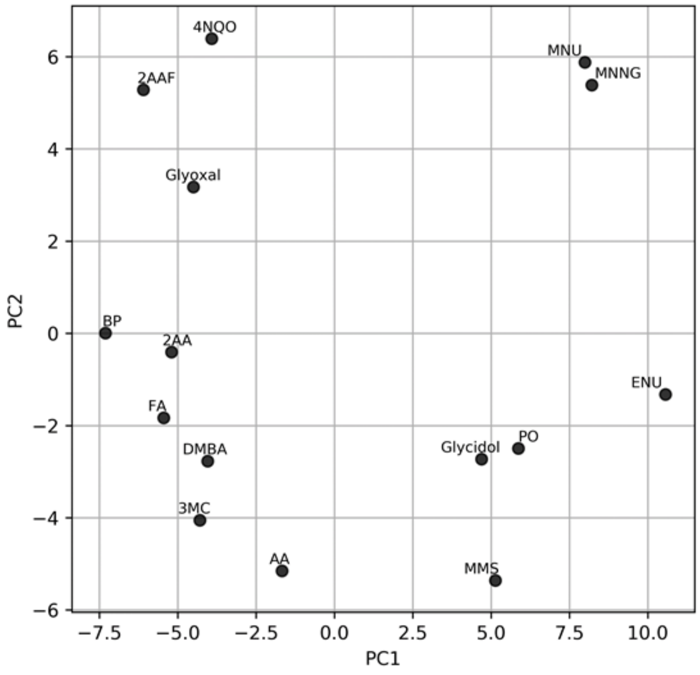

Fig. S4. Principal component analysis results using 96-trinucleotide mutation patterns for each mutagen.

The PCA score plot for each mutagen is based on their PC1 and PC2 values.

(a) (b)

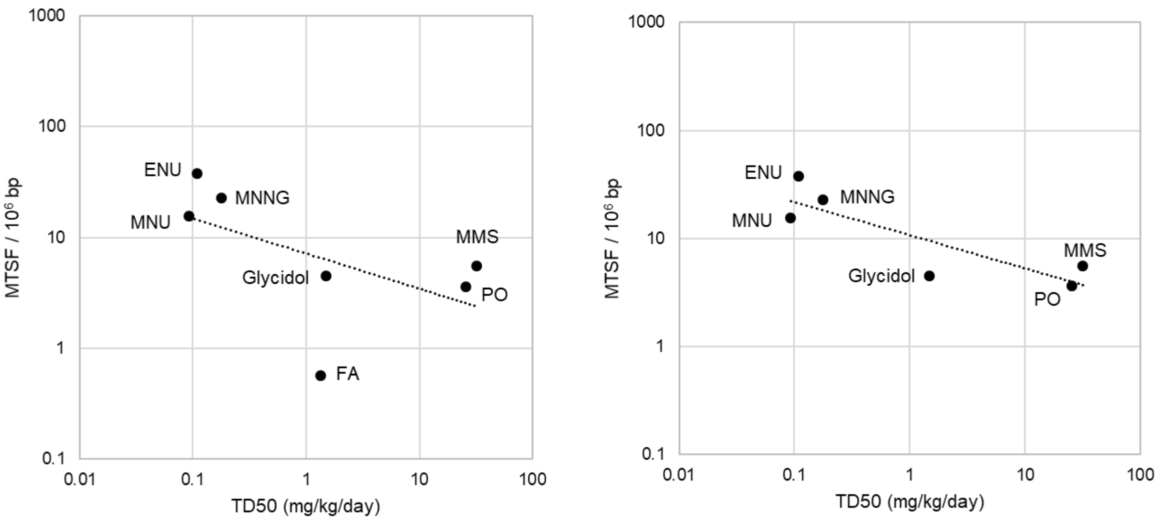

Fig. S5. Comparison between MTSF and TD50 values among direct-acting mutagens. The total BSs frequencies per 10^6 G:C and A:T base pairs are shown (n = 3). **a** Data showing low correlation (R^2 = 0.301). **b** The data was re-evaluated after excluding FA results; a substantial negative correlation was ~~found~~ observed between MTSF and TD50 values (R^2 = 0.733).

(a)

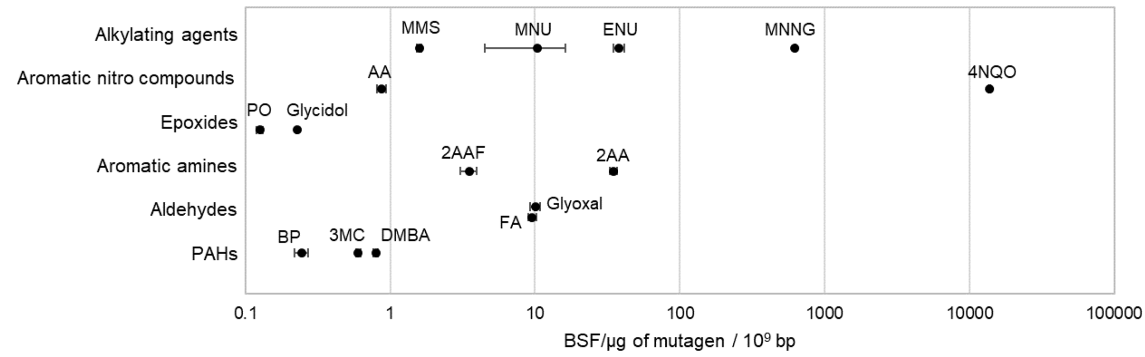

(b)

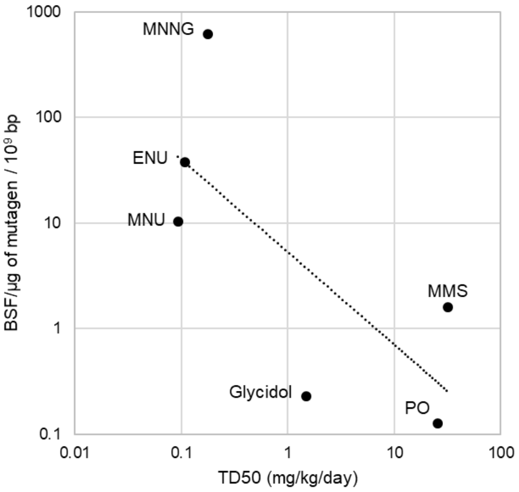

Fig. S6. a The BSF per microgram of mutagen are displayed in log-scale (n = 3). Error bars represent standard deviation. The BSFPD of each mutagen differed among mutagens in the same structural groups. **b** Comparison between the BSFPD and the TD50 values among direct-acting mutagens. BSFPD indicated relatively weak correlation to TD50 values ($R^2 = 0.53$) compared to MTSF.
